# Software Bug Detection Causes a Shift From Bottom-Up to Top-Down Effective Connectivity Involving the Insula Within the Error-Monitoring Network

**DOI:** 10.3389/fnhum.2022.788272

**Published:** 2022-03-07

**Authors:** Joao Castelhano, Isabel C. Duarte, Ricardo Couceiro, Julio Medeiros, Joao Duraes, Sónia Afonso, Henrique Madeira, Miguel Castelo-Branco

**Affiliations:** ^1^Coimbra Institute for Biomedical Imaging and Translational Research (CIBIT)/ICNAS, Faculty of Medicine, University of Coimbra, Coimbra, Portugal; ^2^CISUC-Centre for Informatics and Systems, University of Coimbra, Coimbra, Portugal; ^3^Coimbra Polytechnic—ISEC, Coimbra, Portugal

**Keywords:** error-monitoring, fMRI, insula, connectivity, computer science

## Abstract

The neural correlates of software programming skills have been the target of an increasing number of studies in the past few years. Those studies focused on error-monitoring during software code inspection. Others have studied task-related cognitive load as measured by distinct neurophysiological measures. Most studies addressed only syntax errors (shallow level of code monitoring). However, a recent functional MRI (fMRI) study suggested a pivotal role of the insula during error-monitoring when challenging deep-level analysis of code inspection was required. This raised the hypothesis that the insula is causally involved in deep error-monitoring. To confirm this hypothesis, we carried out a new fMRI study where participants performed a deep source-code comprehension task that included error-monitoring to detect bugs in the code. The generality of our paradigm was enhanced by comparison with a variety of tasks related to text reading and bugless source-code understanding. Healthy adult programmers (*N* = 21) participated in this 3T fMRI experiment. The activation maps evoked by error-related events confirmed significant activations in the insula [*p*(Bonferroni) < 0.05]. Importantly, a posterior-to-anterior causality shift was observed concerning the role of the insula: in the absence of error, causal directions were mainly bottom-up, whereas, in their presence, the strong causal top-down effects from frontal regions, in particular, the anterior cingulate cortex was observed.

## Introduction

Software programming, in particular, the task of code reviewing is a complex and relatively recent human activities, involving the integration of mathematical skills, recursive thinking, language processing, and error-monitoring (Fedorenko et al., 2019). The study of these skills from a neuroscientific perspective has received an increasing interest. However, the neural underpinnings of source-code programming skills are still a matter of debate, in particular, in which it concerns causal networks. There are recent studies on brain regions addressing code understanding (Siegmund et al., 2014a; Castelhano et al., 2019; Ivanova et al., 2020; Peitek et al., 2020). However, the important cognitive component of programming related to software bug detection that in neuroscientific terms can be seen as an error-monitoring process has not been considered previously. In this line, the studies that work on this topic focus on syntax errors (Siegmund et al., 2014b) or comparisons with other well-known human skills (e.g., math and reading) (Hassenfeld et al., 2020) but did not address code inspection in the context of bug detection moments. In this study, we probed in-depth program understanding by requesting participants to identify bugs in a program unit, reproducing a code inspection drill (Siegmund et al., 2014a; Castelhano et al., 2019), a sort of task, which is heavily used in the software industry. This is a more challenging task than conventional program reading and comprehension task, as finding a bug requires a more in-depth program understanding (Couceiro et al., 2019). We thus aimed to investigate the general neural correlates of decision-making during source-code understanding and, in particular, the role of the insula, from a causal perspective, in the network of error-monitoring, during bug detection.

Parts of the insula have been reported as being associated with emotional processing, memory, decision, and sensory regulation in relation to autonomic control (Marxen et al., 2021). This region is an important part of the salience network, which not only drives attention toward target stimuli but also relates to decision-making and error-monitoring (Droutman et al., 2015; Lamichhane et al., 2016; Bastin et al., 2017; Billeke et al., 2020). Our previous study suggests that the insula is activated for challenging deep-level analysis of code inspection and demanding error-monitoring (Castelhano et al., 2019; Medeiros et al., 2021). Moreover, the use of mathematical- or reading-related skills to source-code understanding has been a matter of recent studies (Prat et al., 2020; Castelhano et al., 2021). However, the insula-related causal network involved in these tasks is poorly understood and a connectivity approach is needed. We thus hypothesized that the insula is a key causal pivot in the programming network in the brain responsible for the bug detection in the code. We tested for task-dependent reconfiguration of this neural network associated with human error making and bug discovery during software inspection activities with Granger connectivity measures. To achieve this goal, we performed a functional MRI (fMRI) experiment using a broad set of controls for this cognitively demanding task.

## Materials and Methods

### Participants

During the recruitment phase, we screened 49 participants with a screening technical questionnaire: the main objective of this questionnaire was to assess the coding skills of the candidate. This questionnaire was composed of 10 programming questions, scored with 1 point for correct answer and 0 points for incorrect answer. The minimum possible score was 0 points, and the maximum score was 10 points. Only subjects that scored + 4 were included in the neuroimaging study. A group of 21 male participants (one left-handed) were recruited to this experiment. The range of programming experience was 2–15 years (average: 6.71 ± 4.69 years), and the range of the test score was 4–10 (average: 6.29 ± 1.61). The participants were software development professionals with C programming language and code inspection. The participants’ mean age was 25.56 ± 6.85 years, and all participants had normal or corrected-to-normal vision. The study was approved by the Ethics Committee of the Faculty of Medicine of the University of Coimbra, in accordance with the Declaration of Helsinki, and all experiments were performed in accordance with relevant guidelines and regulations. Informed consent was obtained from all participants.

### Error-Monitoring Task

**FIGURE 1:**
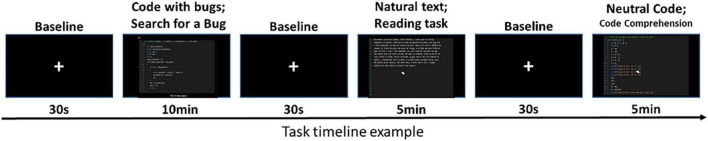
Task timeline. Code with and without bugs and natural text blocks were presented randomly interleaved with a fixation cross for rest. Participants were instructed to search for bugs (error-monitoring/discovery), read the story (Text; control task), and perform a code comprehension task. These stimuli had more than one page, and the participants could navigate the code/text pages with a joystick.

The task included four runs of code inspection. Four different conditions were presented randomly in each of the four runs: Baseline (fixation cross); Natural language reading, i.e., in this task, a text in natural language is presented to the participant; Neutral code (without bugs) snippet reading, i.e., in this task, the participant is presented with a screen containing a simple and iterative C code snippet to be analyzed; Code with bugs (Bug detection), i.e., in this task, a code snippet in C language (Bucket sort; Fibonacci; Hondt method; and Matrix determinant) is displayed to the subject (the subject is asked to analyze and inspect the code for bug (software faults) detection). The task was a replication of our previous study (Castelhano et al., 2019) including additional control conditions to isolate the cognitive components related to bug detection. It was designed to be as close as possible to real-life source-code inspection and bug detection (error-monitoring). Stimuli presentation was implemented with Virtual Reality Toolkit Vizard (WorldViz, Santa Barbara, CA, United States) and displayed in an LCD HD monitor (NordicNeuroLab, Bergen, Norway) placed approximately 156 cm away from the participant’s head (that could be seen through a mirror system mounted above the participant’s head), with a frame rate of 60 Hz. The participants had to identify bugs in the source code and signal the events in the corresponding button of the screen (Figure 1). Participants were able to activate the controls and navigate through the code using an fMRI-compatible joystick (Hybridmojo, San Mateo CA, United States). To be able to evaluate the true error-related decision moments, the participants were also instructed to select a line as soon as they suspected the presence of a bug (“suspicion/eureka moment”) and to confirm the bug in the button “Bug” when they were sure that there was a fault (“Bug detection”). All the behavioral and functional data were recorded simultaneously and synchronized. The stimuli were randomized and counterbalanced between subjects to avoid order effects. The duration of each condition was 30 s for Baseline and was subject-dependent but never exceeds 10 min for the other conditions (Natural text 5 min, Neutral code 5 min, and Code with bugs 10 min). In this line, each experimental block (four codes with bugs; four neutral codes; four texts) ends when the subject decides to press the “End” button. Performance measures were calculated per subject using the following equations: Sensitivity = TP/(TP + FN); Precision = TP/(TP + FP); TP, True Positive; FP, False Positive; FN, False Negative and Spearman’s correlation analysis was calculated between these measures and subjects’ screening score, years of experience, and brain activation data.

### Functional MRI Acquisition Parameters

Brain imaging structural and functional data were acquired in a 3T Prisma MRI scanner (Siemens, Erlangen, Germany) with a 20-channel head coil. Anatomical high-resolution (1 mm^3^) isotropic images were acquired using MPRAGE sequence [repetition Time (TR) 2,530 ms, echo time (TE) 3.5 ms]. The anatomical data of each participant were used for further co-registration with the functional data. For brain activation maps, echo planar imaging (EPI) sequences were acquired with a slice thickness of 3 mm and voxel size of 4 mm^2^, 45 slices covering the whole brain, with a repetition time of 3,000 ms, an echo time of 30 ms, a flip angle of 90°, a matrix size of 64 × 64, and a field-of-view of 256 × 256.

### Functional MRI Preprocessing

The fMRI analysis was performed using BrainVoyager 22 (BrainInovation). The preprocessing was performed with the default parameters (cubic spline slice scan time correction; trilinear 3D motion correction). Structural and functional data were co-registered and transformed to the standard Talairach space. A random-effects general linear model (Friston et al., 1995) multistudy/subject analysis was then performed to assess the brain activity patterns of bug detection moments (suspicion and bug confirmation). Each of these “detection” events had a duration of 3 s before the button press by the subject, *N* = 199 suspicion events and *N* = 114 bug confirmation events. Each condition block was also used as a predictor for further analysis (Baseline, Natural text reading, Neutral code without bugs, and Code with bugs). Each subject had 100 images (TRs) of baseline during the experiment. The median number of images was 173 (min: 79; max: 200 TRs) for the code, 33 (min: 16; max: 79) for the neutral, and 36 (min: 14; max: 47) for the text conditions. There was unbalance duration in these condition predictors, but this was taken into account in the statistical analysis by using a covariate representing the duration of each predictor. Data were corrected for multiple comparisons with the family-wise error (FWE) approach at the single voxel level (*p* < 0.05).

### Functional MRI Connectivity Analysis

The main goal of this study was to characterize the role of the insula as a key node of the error-monitoring network during source-code understanding (Sridharan et al., 2008). Based on our previous study (Castelhano et al., 2019), we used the predictor of the bug suspicion moments to define a region of interest (ROI) in the insula for each participant. We then calculated the Granger causality map (GCM plugin tool from BrainVoyager with the default parameters, *Alard Roebroeck*, version 16) to explore the connectivity of this region for the four different conditions. We used this plugin to find the regions that influence/are influenced by the insular ROI. We chose to compute GCM because it is a method that makes it possible to explore instantaneous and directional influences between regions without an *a priori* model (without a directed graph model of assumed regional connections). In short, a GCM is computed with respect to a single selected reference region and maps both sources of influence to/from the reference region (we used the insula as seed). We used this as an exploratory technique to map if the connections change with experimental conditions. Such a comparison strategy removes common nuisance factors. We set a series of preprocessing steps to overcome GCM limitations (Stokes and Purdon, 2017). This includes slice scan time correction before 3D motion correction. Additionally, the vector autoregression (VAR) models used in the computation of GCMs are needed for temporal stationary time series; in this line, we removed linear trends and slow-wave components at the voxel level by linear trend removal (LTR) and temporal high-pass filtering (THP). Finally, we compared the resulting maps computed over different conditions for the same reference region because Granger causal influence in a directed Granger Causality map (dGCM) can be assigned to interactions between neuronal populations if that influence can be shown to be modulated by experimental conditions (Seth et al., 2015; Barnett et al., 2018). Using this plugin, it was possible to compute GCM and identify the sources or targets of information of the selected ROI. In brief, it uses the Granger principle to estimate if the information from the past in a time series may improve the prediction of a current value of a second time series (Roebroeck et al., 2005). With this approach, we computed the insular effective connectivity maps (directed functional connectivity) for each condition. The positive value represents voxels where influence from the reference region dominates (i.e., those are the targets of the ROI), and the negative value at voxels signal the voxels where influence to the reference region dominates (i.e., those are the sources of influence to the ROI). Thresholds for the connectivity maps were computed by non-parametric bootstrap procedures (5,000 surrogate simulations). The false discovery rate (FDR)-based procedures were used on the bootstrapped *p*-values to give thresholds that are adjusted for multiple comparisons within the map (*p* < 0.01).

For the sake of comparison, we overlaid the maps of each direction (influence to/from insula) and condition and exported the results in Supplementary Table 1 of region coordinates.

## Results

We performed an fMRI experiment to study for the first time the task dependence of effective connectivity of the insula as evoked by a software bug detection task within the error-monitoring network. Our study included a set of control conditions to directly address the role of the insula specifically related to error-monitoring during source-code comprehension and searching for a bug as compared to natural text reading or neutral code comprehension without bugs.

**FIGURE 2:**
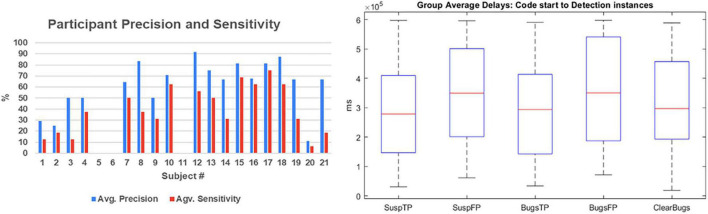
Summary of behavioral data. Subjects’ accuracy reported as precision and sensitivity measures are reported in the left plot. Notably, subjects 5 and 6, as well as subject 11, did not report any bugs; thus, we were not able to calculate their parameters. The box plot on the right has shown the average delays from code start to the eureka moments. No significant differences were found between events. TP, true positive; FP, false positive.

For the sake of testing group homogeneity, we defined two subgroups of experts (screening test > 6; *N* = 12) vs. less-expert participants (screening test < 6; *N* = 9). We performed a non-parametric homogeneity test (Mann–Whitney *U* test) that revealed that the distribution of brain activation is the same across categories (experts vs. less-expert participants): Mann–Whitney *U* test (18) = 46; *p* = 0.594; the distribution of years of experience (*p* = 0.462), the distribution of age (*p* = 0.385), and precision (*p* = 0.247) are the same across categories of expertise. These data revealed a homogeneous group of programming experts that could reliably perform code inspections. Precision was above 62% on average (Figure 2). Furthermore, we did not find differences in the delay of the detection events (Figure 2) that could be used to predict the outcome of the decision (e.g., if it is an FP or TP report of a bug). We performed a correlation analysis between the brain activation and the accuracy measures, and no significant correlation was found (*r*^2^= 0.09). In contrast, we found a significant Spearman’s rho correlation of 0.5 (*p* = 0.034) between the screening score and the performance measure of precision, suggesting that the behavioral data may be a result of experience, but no significant correlation was found between the years of experience of the subjects and the performance or the brain activity results (*r* = 0.486, *p* = 0.078).

**FIGURE 3:**
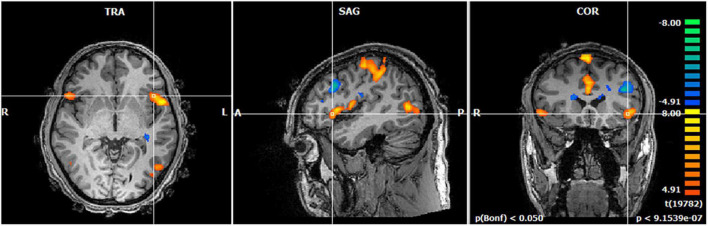
Brain activation map of bug detection when searching for bugs. The regions that are activated at the first insight of bug detection are shown (eureka moment of suspicion) at the group level. We found significant activation in the anterior insula replicating our previous study (Castelhano et al., 2019).

The activation maps for the contrast of the Code with bugs vs. Neutral code (code without bugs) revealed the set of areas responding to the activity of searching for a bug during source-code understanding. In particular, we found activation in the bilateral anterior insula [*t* = 4.98; *p*(Bonferroni) < 0.05] which is a pivotal hub within the salience network, in error-monitoring tasks, in particular, during bug monitoring. This insula activation was even more evident at the “eureka” moment of suspicion when the subject detects a bug in the code (Figure 3).

**FIGURE 4:**
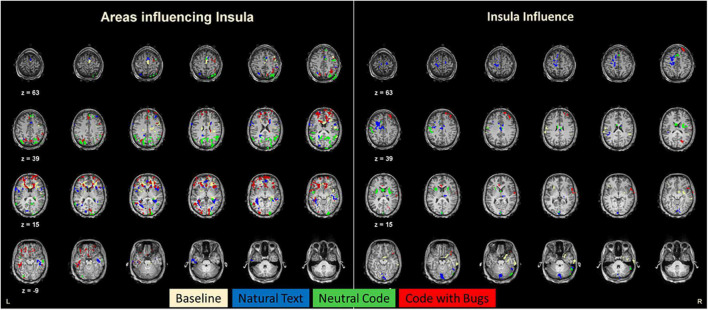
Connectivity maps for the insula. The left panel represents the brain (Granger) connectivity map of regions that give input to the insula. The right panel shows the brain regions receiving input from the insula. In these maps, each color map represents each condition of the experiment. Notably, the “Code with bugs” condition recruits a larger set of areas to integrate with the insula, and these are located in more anterior regions of the brain compared to the other conditions that have more posterior connections. In sum, bug detection leads to an anterior shift of influences.

To investigate the effective connectivity of this insular area during this programming task, we calculated Granger connectivity maps and found the regions that causally influence the insula or that are influenced by the insula (ROI). These results show a striking condition dependence of effective connectivity (Figure 4). For the sake of comparison, Figure 3 represents these maps in different colors for each condition. Regarding the natural text reading condition, the insula is mainly influenced by visual, parietal, frontal, and ventrotemporal regions known to be activated during reading tasks (Dehaene et al., 2015). Furthermore, for the neutral code condition, the regions with connections with the insula closely match the posterior, visual, dorsal, and ventral streams. In contrast, for the code with bugs, when the participants search for bugs, a set of anterior/frontal decision-related regions show a causal relation with insular activity. These areas include Brodmann area (BA) 6 and 8, which are the medial prefrontal cortex regions involved in memory, reasoning, error detection, or attention (Iannaccone et al., 2015), and most importantly, the regions of the anterior cingulate cortex (ACC) (BA 24 and 32), which are the well-known regions of the error-monitoring circuitry (Gilbertson et al., 2020; Roe et al., 2021). This suggests a posterior-to-anterior shift of causal influences to the insula when error detection is required.

Taken together, our results suggest that the insula is a pivotal hub concerning reconfiguration of the directionality of the interactions as it becomes the target for an anterior shift of causal influences from frontal error-monitoring and executive function regions when bug detection is required. This region is driven mostly by posterior regions for the natural text reading and controlled by ACC when a very complex task of searching for bugs is required, connecting with frontal regions related to decision-making and error-monitoring within the salience network.

## Discussion

The main hypothesis of this study was that the insula has a pivotal role during error-monitoring processes, in particular, in relation to software bug detection. Furthermore, this study provides a neuroscientific perspective on cognitive aspects influencing software reliability. Our correlation analysis of the behavioral results with the subject’s experience confirmed an effect of experience, as more accurate participants are the more experienced. Nevertheless, no correlations were found between performance, experience, and brain activity, and we have shown that our group is homogeneous relatively to years of experience, age, and performance precision.

Our study directly addressed the role of the insula in error-monitoring during source-code comprehension during different types of trials (including code with bugs, neutral code, and natural text). This region is a novel key hub since the importance of other regions related to reading, language, or mathematical processing relevant to the development of the programming skills has been addressed before. Prat et al. (2020) in fact, suggested a framework for understanding programming aptitude, in relation to reading skills. In contrast, our previous review suggested a weighted use of reading and mathematical skills for programming (Castelhano et al., 2021). We extended these studies by studying the role of the insula in source-code understanding and other control tasks within the framework of a connectivity hypothesis. We confirmed our hypothesis by demonstrating condition-dependent effective connectivity of the insula. This provides a new perspective to previous studies that reported increased error-related activity in a network of cortical regions that include ACC, dorsolateral prefrontal cortex, inferior parietal lobe, and anterior insula (Gilbertson et al., 2020). However, if it is the insula or, for example, the dorsal anterior cingulate cortex (dACC) that can be seen as a central hub of this network is still a matter of debate (Bastin et al., 2017). Our results suggest that the latter provides directed influences to the former when error-monitoring is increased. Our previous study and other recent publications showed that insula activity is correlated to behavioral performance and reaction times in distinct tasks (Castelhano et al., 2019; Marxen et al., 2021). Evidence that the insular cortex also seems to be involved in performance monitoring (Bastin et al., 2017; Billeke et al., 2020) and our Granger causality findings showing that error-monitoring-related activity triggers a shift of causal influences to frontal regions, in particular, ACC, make a strong basis toward the notion that the insula is a pivotal region during error-monitoring. Future studies should elucidate how this anterior reconfiguration of connectivity generalizes to other tasks, in children and adults’ learning (Roe et al., 2021).

Using GCM can be a limitation of the study as there is some ongoing discussion on the assumptions that should be met for it to be used in neuroscience research (Stokes and Purdon, 2017; Barnett et al., 2018). Nevertheless, this is well-suited to the exploratory analyses to map if the connections change with experimental context (comparison between conditions wherein hemodynamic response functions (HRFs) can be assumed to remain unchanged) (Friston, 2011; Seth et al., 2015) as we performed in this study. Moreover, we performed a series of preprocessing steps that minimize the possible bias and make this approach a conceptually satisfying and statistically powerful method for directed functional connectivity analysis of mapping influences between an ROI and the rest of the brain (Roebroeck et al., 2005). A Granger causal influence in a dGCM can be assigned to interactions between neuronal populations, if that influence can be shown to be modulated by experimental conditions. In this line, we found a clear role for regions within the salience networks in error-monitoring during challenging bug discovery tasks. The insula (BA 13) becomes a connectivity target region from frontal regions (including ACC) during cognitively demanding error-monitoring tasks such as code review. The elucidation of this anterior shift and neural mechanisms underlying error detection during code inspection may also be quite relevant for the software industry.

## Data Availability Statement

The raw data supporting the conclusions of this article will be made available by the authors, without undue reservation.

## Ethics Statement

The studies involving human participants were reviewed and approved by Comissão de Ética Universidade de Coimbra. The patients/participants provided their written informed consent to participate in this study.

## Author Contributions

JC designed the experiment, acquired the data, analyzed the data, interpreted the results, and wrote the manuscript. ID developed the paradigm, analyzed the data, and revised the manuscript. RC designed the experiment, acquired the data, and revised the manuscript. JM acquired the data and revised the manuscript. JD designed the stimuli for the task, acquired the data, analyzed the behavioral data, and revised the manuscript. HM was the PI of the BASE project that provided funding for the study, designed the experiment, acquired and analyzed the data, and wrote the manuscript. MC-B supervised all the process of experimental design, data acquisition, and analysis, interpreted the results, and wrote the manuscript. All authors contributed to the article and approved the submitted version.

## Conflict of Interest

The authors declare that the research was conducted in the absence of any commercial or financial relationships that could be construed as a potential conflict of interest.

## Publisher’s Note

All claims expressed in this article are solely those of the authors and do not necessarily represent those of their affiliated organizations, or those of the publisher, the editors and the reviewers. Any product that may be evaluated in this article, or claim that may be made by its manufacturer, is not guaranteed or endorsed by the publisher.
